# How the misincorporation of ribonucleotides into genomic DNA can be both harmful and helpful to cells

**DOI:** 10.1093/nar/gku773

**Published:** 2014-08-26

**Authors:** Catherine J. Potenski, Hannah L. Klein

**Affiliations:** Department of Biochemistry and Molecular Pharmacology, New York University School of Medicine, New York, NY 10016, USA

## Abstract

Ribonucleotides are misincorporated into replicating DNA due to the similarity of deoxyribonucleotides and ribonucleotides, the high concentration of ribonucleotides in the nucleus and the imperfect accuracy of replicative DNA polymerases in choosing the base with the correct sugar. Embedded ribonucleotides change certain properties of the DNA and can interfere with normal DNA transactions. Therefore, misincorporated ribonucleotides are targeted by the cell for removal. Failure to remove ribonucleotides from DNA results in an increase in genome instability, a phenomenon that has been characterized in various systems using multiple assays. Recently, however, another side to ribonucleotide misincorporation has emerged, where there is evidence for a functional role of misinserted ribonucleotides in DNA, leading to beneficial consequences for the cell. This review examines examples of both positive and negative effects of genomic ribonucleotide misincorporation in various organisms, aiming to highlight the diversity and the utility of this common replication variation.

## INTRODUCTION

Faithful replication of the genome is essential for the propagation of cells, and multiple mechanisms have evolved to insure the accurate copying of DNA during cell division. In general, replication fidelity refers to the preservation of base identity between parent and daughter strands of DNA. Replication enzymes insert deoxyribonucleotides (dNTPs) at the 3′ end of the nascent strand that are complementary to the opposite base on the template strand. Proofreading activity of DNA polymerases as well as other, post-replicative enzymes act to recognize and repair mispaired dNMPs that are inserted during replication. In this way, there is multi-tiered protection against mutations, and the error rate of replication is kept extremely low.

Apart from insuring the accurate identity of a complementary base, another facet of replication fidelity is the discrimination of the sugar backbone of the nucleic acid strand (ribose rNTPs versus deoxyribose dNTPs) so that the correct sugar NTP is chosen (1). The two classes have extremely similar structures, differing only by a single OH group on the 2′ carbon of the sugar. Polymerases are challenged with distinguishing between the two types of nucleotides; otherwise, they risk inserting the incorrect substrate into the newly replicated nucleic acid strand. Genomic DNA with rNMPs embedded in it is a non-canonical substrate for processes such as replication, transcription and repair and must itself be processed in order for basic DNA metabolism to proceed smoothly (2–4). Also, rNMPs render the DNA backbone more labile, affecting its stability (5). As such, replication inaccuracy in the form of misincorporated ribonucleotides has the potential to be as problematic for the cell as a base pair change mutation. However, ribonucleotide misincorporation represents the most common type of replication error and occurs quite frequently in normal cells (3,6). This degree of frequency suggests that ribonucleotides in DNA may not be so burdensome as initially thought, or even that they provide an advantage. The subject of this review is the misincorporation of rNTPs into genomic DNA, and the consequences, both positive and negative, that follow from this.

### Ribonucleotide misincorporation occurs frequently

The failure of DNA polymerases to perfectly discriminate between rNTPs and dNTPs leads to the misinsertion of ribonucleotides (1). Rates of ribonucleotide misincorporation are influenced by a combination of many factors, including the replicative polymerase being used, the rNTP in question, the surrounding sequence context and the ratios of rNTP to dNTP pools. Even in normal cells, ribonucleotide misincorporation rates can be quite high (3,6). Although the DNA polymerases can remove rNMPs though the proofreading function (6,7), this is not efficient enough to prevent significant levels of rNTPs from remaining incorporated into the DNA backbone. Fundamentally, keeping ribonucleotides completely out of the genome is impossible, and potentially, not even desirable.

Intracellular nucleotide pool levels often change, being subject to cell-cycle stage, nutrient amounts and oxidative stress, among other factors (3,8). There is a high demand for rNTPs during active transcription, and the default state of the nucleus is to have much greater levels of rNTPs than dNTPs. The dNTP concentration range estimated from a yeast cell is 12–30 μM, while the rNTP concentration range is up to two orders of magnitude higher, at 500–3000 μM (3). Therefore, the correct substrate for DNA polymerases is found in much lower concentrations than a highly similar, yet incorrect substrate. As such, sugar discrimination is a very important trait for DNA polymerases in this environment enriched for rNTPs over dNTPs.

DNA polymerases have evolved ways to discern between these two very similar substrates (1). Powerful *in vitro* studies shed light on the mechanism of sugar discrimination as well as the different rates of misincorporation by different DNA polymerases from bacteria (9), yeast (3) and humans (6). *Escherichia coli* DNA polymerase I was capable of incorporating rNTPs, with rCTP being incorporated the most frequently and rUTP the least (9). Misincorporation rates were determined for the yeast leading strand polymerase ϵ (Pol2) and the lagging strand polymerase α (Pol1) and polymerase δ (Pol3) for each rNTP (3). There is quite a substantial range in misincorporation rates, with Pol1 having the lowest discriminatory abilities and Pol3 having the highest. Based on these rates, Nick McElhinny *et al.* estimate that greater than 10 000 rNMPs can be misincorporated into the yeast genome during one round of replication. Estimates of individual polymerase rNTP incorporation rates have shown that Pol2 (polymerase ϵ) is more rNTP-permissive than Pol3 (polymerase δ) (3). *In vitro* studies of the human Polδ determined that the enzyme misincorporates ribonucleotides at a rate of ∼1 rNTP per 2000 dNTPs, resulting in >1 000 000 rNMPs embedded in DNA after one cycle of replication (6). These estimates based on *in vitro* rate determinations are supported by *in vivo* data. In mammalian cells deficient for RNase H2, where rNMPs are not efficiently removed from the genome, the ribonucleotide content of genomic DNA was assayed through alkaline gel analysis and was estimated to be about 1 000 000 rNMPs, agreeing well with the *in vitro* approximations (10,11). Genomic rNMPs, therefore, represent by far the most common type of DNA aberration.

Although much is known about global levels of misincoporated ribonucleotides, there is little information about where precisely these misinsertions are occurring. Whether it is an entirely stochastic process or there are sequence hot spots where a ribonucleotide is more likely to be inserted into the genome remains unclear, although position specificity is seen *in vitro* (3). Hot spot ribonucleotide insertion sequences could be variable, changing with environment, developmental stage or cell type. They could be context and/or sequence dependent. Whole-genome studies about where and when these misinsertions occur will be very enlightening and lead to a deeper understanding of the consequences of ribonucleotide misincorporation.

### Ribonucleotides change the character of DNA

**Figure 1.**
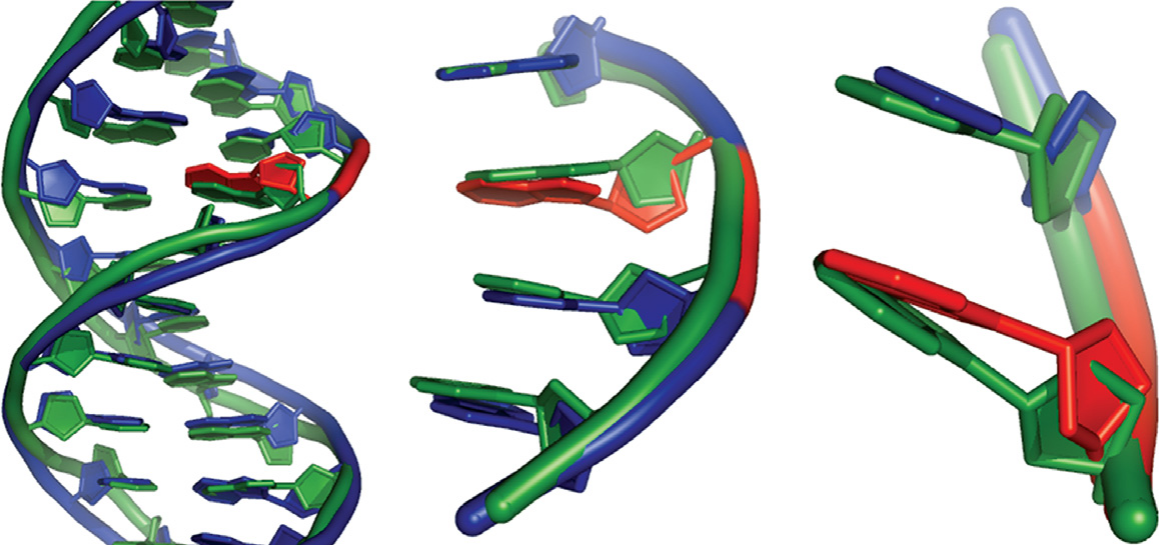
Structure of DNA with an embedded rNMP. Aligned structures of 12 nt DNA molecule (PDB ID 436D; green) and a DNA molecule (PDB ID 2L7D (13); blue) with an embedded ribonucleotide (red).

Having a ribonucleotide inserted into DNA sensitizes the backbone to cleavage due to the higher reactivity of the extra 2′OH-containing rNMP (5). Therefore, the stability of the DNA molecule can be diminished by the presence of ribonucleotides. Additionally, the atypical rNMP residues can alter the shape of the DNA molecule. The physical structure of a single ribonucleotide embedded in DNA has been explored through crystallography and nuclear magnetic resonance (12,13). The structure of a 12 bp double-stranded DNA fragment with a single internal rGMP was compared with that of the same fragment containing only dNMP residues. Superimposition of these two structures reveals deviation in base angles and backbone shape in the molecule with the embedded ribonucleotide versus the intact DNA (Figure 1). But, the overall B-form DNA structure is preserved, demonstrating that a single ribonucleotide does not impart overly severe distortions to the DNA (13). However, the deviation is still such that it is capable of impeding the progression of the replication machinery (2–4). Similarly, the proper assembly of nucleosomes is negatively impacted by ribonucleotides in the genome, where having 5% or greater ribonucleotide content in DNA abolishes nucleosome formation (14). Moreover, a single ribonucleotide embedded in duplex DNA can result in helix perturbation at the RNA:DNA base stack which can alter protein recognition and binding (15,16). Therefore, in order to avoid the disruption of these central cellular processes, it is important for misinserted ribonucleotides to be removed from the genome.

### Removal of ribonucleotides embedded in DNA is catalyzed by RNase H2 enzymes

**Figure 2.**
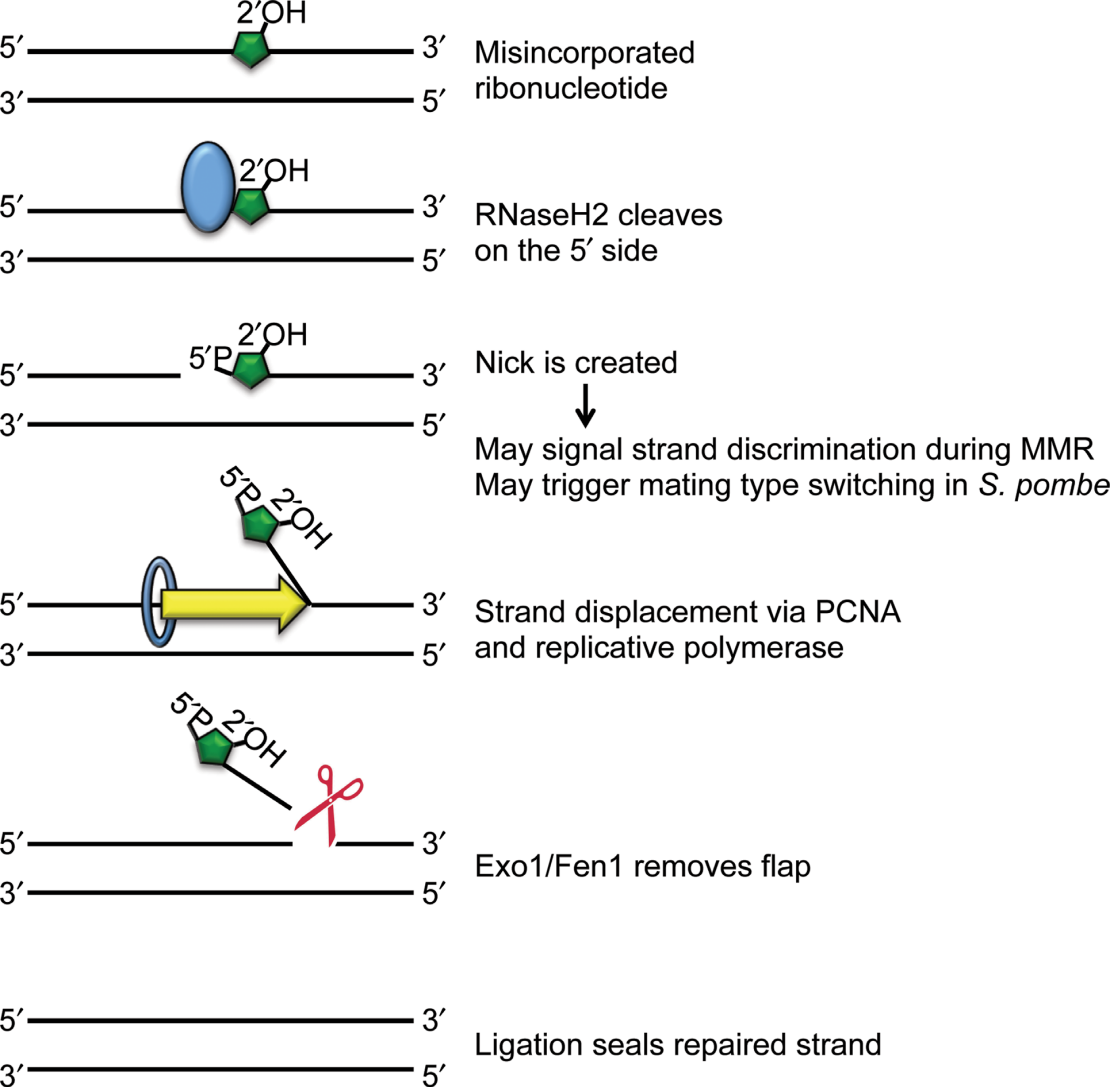
RER model. A misincorporated ribonucleotide (green) is recognized by RNase H2 (blue), which cleaves on the 5′ side, leaving a nick. PCNA (blue ring) and DNA polymerase (Polϵ or Polδ) displace the strand with the ribonucleotide by replicating new DNA. A 5′ flap endonuclease (red) cuts, releasing the ribonucleotide-containing DNA strand. DNA ligase I seals the remaining nick, resulting in fully repaired DNA. The creation of the nick may signal strand discrimination or mating type switching, highlighting potential beneficial roles of rNTP incorporation.

The enzyme responsible for the removal of ribonucleotides from DNA is RNase H, which hydrolyzes the RNA component of DNA:RNA hybrids and is preserved in all kingdoms of life (17). There are two main classes, RNase H1 and RNase H2 (18), which have very divergent sequences but share similar structural domains and enzymatic mechanism (19). RNase H1 enzymes recognize a DNA:RNA hybrid of at least three residues, and therefore can remove extended DNA:RNA hybrids, or R-loops, but cannot catalyze the removal of a single rNMP embedded in DNA (20). In contrast, the processing of misincorporated ribonucleotides is undertaken by RNase H2 (Table 1), which cleaves on the 5′ side of an rNMP that is found in a DNA context (21), initiating the removal of the residue and the faithful repair of the site in a process termed ribonucleotide excision repair (RER) (22) (Figure 2).

**Table 1.** RNase H2 enzymes. Summarized are the characteristics of RNase H2 enzymes from bacteria, yeast and mammals.

	Bacteria	Yeast	Mammals
RNaseH2	RnhB	Rnh201	RNaseH2A
		Rnh202	RNaseH2B
		Rnh203	RNaseH2C
Null mutant	Viable	Viable	Inviable
Increased genome instability	No	Yes	Yes
Recruitment	Unknown	PIP-box	PIP-box

RNase H2 also likely has a role during replication to help remove Okazaki primers on the lagging strand (23). In yeast, the genes that encode the three RNase H2 subunits are up-regulated during S phase (24), and deletions of RNase H2 genes are synthetically lethal or show synthetic sickness/slow growth with deletions of other genes that have crucial functions in replication, including Rad27 (Fen1). Additionally, RNase H2 has a PCNA-interacting peptide domain (PIP-box) on one of its subunits. PCNA was co-crystallized with RNase HII from *Archeaoglobus fulgidus*, and human PCNA was co-crystallized with a peptide from the RNase H2B subunit containing the PIP-box (25). These structural data revealed interaction between RNase H2 and PCNA. *In vivo* assays were also performed, showing the recruitment of RNase H2 to DNA by PCNA. The PCNA interaction is thought to allow RNase H2 to perform its roles during replication in Okazaki fragment processing and during RER. Indeed, recently, nascent chromatin capture and mass spectrometry analysis showed that the RNase H2 complex is enriched at replication forks in human cells (26). Altogether, these data point to a role for RNase H2 during replication, suggesting that RER is a fast process that is closely coupled to replication fork progression.

### Consequences of RNase H2 impairment

#### Bacteria

Bacteria have monomeric RNase HII enzymes that are more distributive than their eukaryotic counterparts, perhaps because they lack accessory subunits. How the enzyme gets recruited to its substrate remains unknown. In *E. coli*, the Pol III replicative polymerase misincorporates one rNMP residue approximately every 2.3 kb, resulting in ∼2000 rNMPs per newly replicated chromosome (27). The replication machinery is impeded by rNMPs embedded in the template DNA and when there is a high rNTP/dNTP ratio, replication rate is slowed due to competition for proper polymerase substrate (27). Misincorporated ribonucleotides are removed from the *E. coli* genome by RNase HII-mediated RER (28), and it has been shown that mismatch repair (MMR) (29) and nucleotide excision repair (NER) (30) also, to varying degrees, play backup roles in removing rNMPs, particularly when the bases are mispaired.

In *E. coli*, *rnhB* encodes for RNase HII, while *rnhA* encodes for RNase HI. Deletion of *rnhB* does not result in any severe phenotypes and does not lead to an enhanced spontaneous mutation rate (27), consistent with the idea that MMR and/or NER serve as alternative ribonucleotide removal pathways. To learn more about bacterial tolerance of misincorporated rNMPs, work was done using *E. coli* translesion DNA polymerase pol V, which is highly permissive to ribonucleotide incorporation (31). Using a point mutant that essentially abolishes sugar discrimination altogether, resulting in extremely high levels of misincorporated ribonucleotides, it was shown that *rnhB*, *rnhA* and NER factors are capable of removing even a very high load of rNMPs, with minimal contribution from MMR or base excision repair factors (30). In this background, where ribonucleotides are misinserted to such a high degree, there are likely to be stretches of multiple rNMP residues, not exclusively single misincorporated ribonucleotides, so *rnhA* seems to act redundantly to *rnhB*. The NER pathway most likely acts as a backup mechanism for clearing ribonucleotides in the genome that engages when the primary, *rnhB*-mediated pathway is compromised.

#### Yeast

RNase H2 is comprised of three proteins: catalytic subunit Rnh201 and accessory subunits Rnh202 and Rnh203 (32). Rnh202 and Rnh203 create a stable complex that then interacts with Rnh201 to form a catalytically active enzyme. Although the exact role of Rnh202/Rnh203 is unknown, they most likely act to stabilize the complex, contribute to processivity or interact with other factors that are important for the function or recruitment of the enzyme. A PCNA-interacting motif (PIP box) is located on the C-terminus of the Rnh202 subunit, which is likely to promote recruitment of the complex to the replication fork, as mutation of the PIP box in the human RNASEH2B subunit disrupts colocalization to replication foci (25). All three yeast subunits are essential for function, as deleting any of the three results in a null phenotype (33,34).

RNase H2 deletion mutants are viable and not sensitive to agents of DNA damage, but inactivation of RNase H2 enzymes has been associated with increased genome instability in yeast (35–37), suggesting that failure to remove ribonucleotides from DNA harms the cell. More specifically, it has been shown that in the absence of RNase H2, harmful, alternative pathways of ribonucleotide removal engage, resulting in replication stress, mutation rate increase and genome instability (37–39).

Based on studies in yeast, the main alternative pathway that catalyzes the removal of ribonucleotides from DNA when RNase H2 enzymes are absent or inactive is a Topoisomerase I (Top1)-dependent mechanism (37). Top1 normally cuts one strand of DNA, creating a transient covalent protein-DNA linkage and allowing for the relief of supercoiling. When Top1 cleaves at a ribonucleotide, the covalent linkage is now susceptible to nucleolytic attack by the 2′ hydroxyl group of the rNMP residue. Top1 is removed, leaving a nick that is flanked by a 2′-3′ cyclic phosphate end and a 5′-OH end (40). In this way, Top1 acts as an endonuclease. Top1 processing of ribonucleotides in the absence of RNase H2 leads to genome instability in the form of increased mutation rates (37,41), increased recombination rates (42,33) and increased chromosome instability (43). RNase H2 mutants have the specific mutation signature of an increase in slippages in short dinucleotide repeats regions. These phenotypes are dependent on Top1 (37,38). It is unclear if the same Topoisomerase I-dependent mechanism acts in mammalian cells. However, genome instability is a hallmark of RNase H2-deficient mammalian cells (10), suggesting that this mutagenic, alternative processing pathway could be conserved.

**Figure 3.**
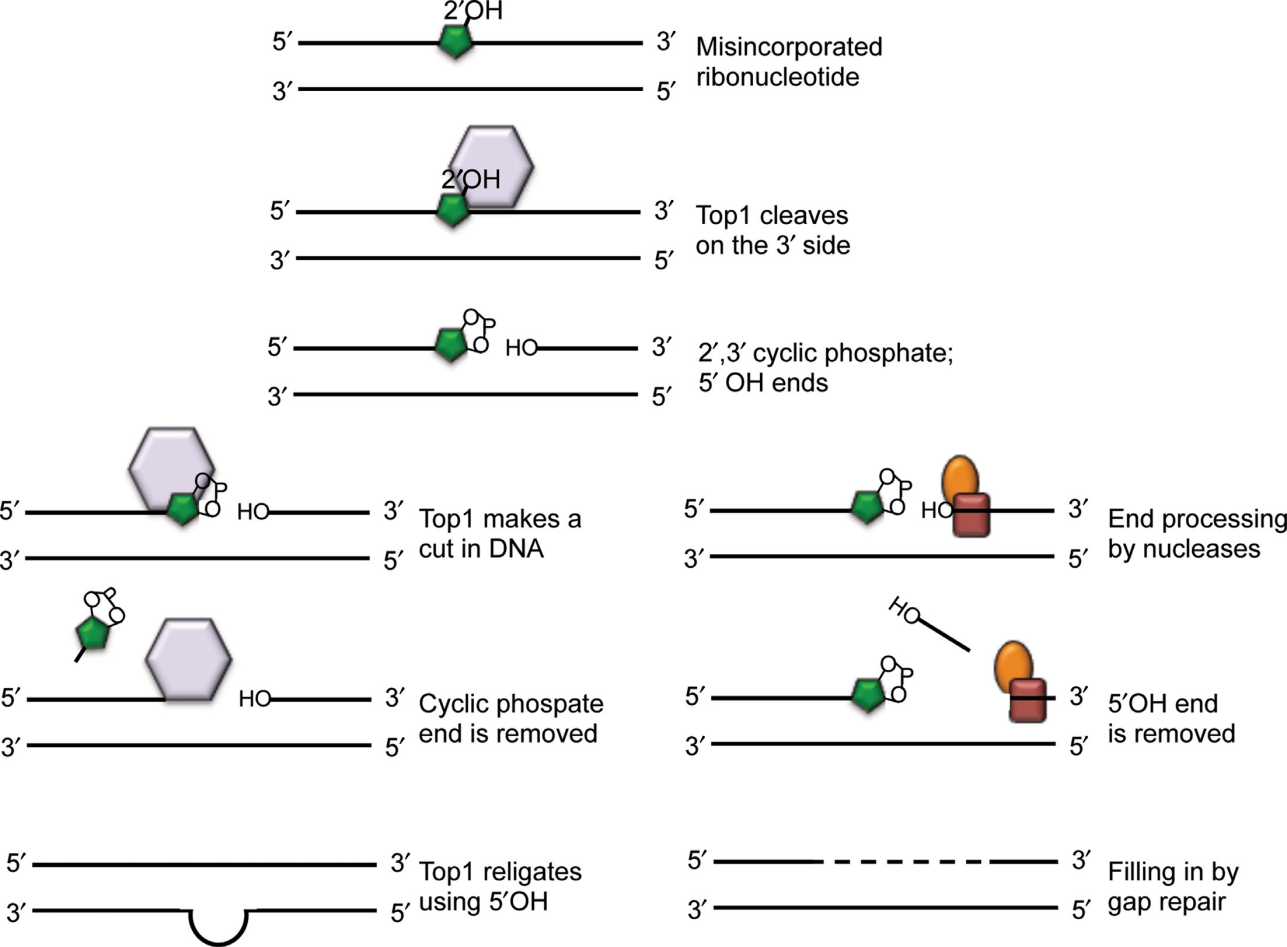
Model of Topoisomerase I-processing of misincorporated ribonucleotides. A misincorporated ribonucleotide (green) in DNA can be cleaved by Topoisomerase I (purple), which cuts on the 3′ side, leaving 2′3′-cyclic phosphate and 5′ OH ends. The lower left panels show processing of the cyclic phosphate end, which could involve Topoisomerase I making a second cut, this time in the DNA, resulting in liberation of a short sequence (2–5 nt) with the cyclic phosphate attached. Religation across a short gap restores intact DNA, but also can lead to mispairing in repeat regions, ultimately seen as slippage mutations. The lower right panels show processing of the 5′OH side via factors that bind at the nick (red, orange), with a 3′-5′ helicase unwinding the DNA and a 5′ flap endonuclease cleaving the displaced strand. This allows for gap repair to synthesize new DNA.

The nick that is created and the non-canonical ligation ends that are left by Top1 cutting at an embedded ribonucleotide likely contribute to the observed genome instability phenotype of cells that lack RNase H2. Several mechanisms by which these lesions get processed have been proposed (Figure 3). One proposed model is that Top1 makes a second cut, this time in DNA, on the 3′ side of the cyclic phosphate (44). This liberates a small fragment containing the cyclic phosphate and allows Top1 to use the 5′OH substrate to ligate across a small gap (45). If this occurs in repeat regions, it can lead to slippage deletions. Another proposed mechanism describes processing on the 5′OH side of the nick, where a 3′-5′ helicase (Srs2) unwinds the DNA and a 5′ flap endonuclease (Exo1) cleaves the displaced strand (33). This prepares the DNA for gap repair, reducing the likelihood of slippages. There are many other potential processing factors that could work with Top1 or subsequent to cleavage to help remove ribonucleotides in the absence of RNase H2. As some of these factors are conserved, elucidation of these alternate pathways will help to shed light on tolerance of misincorporated ribonucleotides in not only yeast, but higher organisms as well.

A number of studies have utilized DNA polymerase mutants that have altered substrate specificity to ascertain how perturbation of ribonucleotide misincorporation rates affect cells (41). The basis for nucleotide sugar discrimination in DNA polymerase ϵ is a conserved tyrosine residue that acts as a ‘gate’ to sterically occlude the 2′OH group on a ribose sugar (46). Mutations to an adjacent methionine residue can either make the gate more flexible or more rigid, allowing for higher or lower rates of ribonucleotide misincorporation, respectively. RNase H2-deficient cells with the polymerase mutant that incorporates more ribonucleotides, *pol2-M644G*, have very high mutation rates and are sensitive to replication stress in the form of hydroxyurea, which inhibits ribonucleotide reductase and affects dNTP pools (41,39). Indeed, even cells with the *pol2-M644G* mutation and functional RNase H2 had higher levels of ribonucleotides in the genome, indicating that they were not all removed efficiently, most likely due to swamping out the ability of RNase H2 to remove them, and a concomitant increased mutation rate. This suggests that it is not just the absence of RNase H2, but the subsequent harmful alternative processing that leads to deleterious effects. The mere presence of ribonucleotides in the genome at a high enough threshold is enough to cause cellular distress, even with fully functional RNase H2.

#### Mammals

The human RNase H2 enzyme is made up of RNase H2A, RNase H2B and RNase H2C, corresponding to the yeast proteins Rnh201, Rnh202 and Rnh203, respectively. Accordingly, RNase H2A is the catalytic subunit and there is a PIP box on RNase H2B. In humans, the inactivation of RNase H2 causes Aicardi–Goutieres syndrome (AGS) (47). This is a rare, genetic neurological disorder characterized by unchecked inflammatory response in the absence of exogenous stimuli (48,49). Mutations in other genes that encode for proteins that are involved in various aspects of nucleic acid metabolism, including SAMHD1 (50), TREX1 (51) and ADAR1 (52), also have been associated with AGS. It is thought that in patients with these mutations, there is a high level of aberrant nucleic acid because proper DNA and/or RNA processing is compromised. This leads to an interferon alpha (INF-α)-mediated, constitutive inflammatory response that has a potent effect on neurological development and function (53). There is no cure for AGS and although it is clear that an overblown immune response underlies the symptoms, the precise connection between RNase H2 mutants and disease causation remains mysterious. Some of the presenting conditions of AGS have similarity to the phenotypes of lupus erythematosus, and mutations in the *TREX1* gene are associated with AGS and lupus erythmatosus. A recent study of AGS patients has suggested that some of these patients with mutations in RNase H2 subunits have some features of lupus, connecting an innate immune response defect to systemic autoimmunity (54).

The majority (∼40%) of all AGS mutations are found in the non-catalytic RNase H2B subunit (49). When the biochemical activity of AGS mutant RNase H2 proteins was queried, all were indistinguishable from the wild-type enzyme, with the exception of only one (RNase H2A-G37S) that showed partially reduced enzymatic activity (47). This strongly suggests that severe mutations in RNase H2 cannot be tolerated, leading to the idea that RNase H2 is likely essential in humans. This is in contrast to organisms such as yeast and bacteria that can survive without RNase H2. The increased size and complexity of metazoan genomes could account for this difference in RNase H2 requirement for survival. Perhaps complete inactivation of RNase H2 would lead to such a high load of ribonucleotides in the genome that it would be too great a burden for mammals to process.

Although for yeast RNase H2 mutants there is a wealth of *in vivo* and genetic data that have been extremely valuable in terms of elucidating RNase H2 function, unicellular organisms make poor disease models. Until recently, the consequence of RNase H2 inactivation in the cells of mammals has been less well studied, but now mouse models for AGS have been generated: a mutant *Rnaseh2b* allele with an artificial stop codon in exon 7 was knocked in to mice (10), and a complete knockout of *Rnaseh2c* was constructed along with a hypomorphic *Rnaseh2b* allele (11). In both cases, when heterozygous mutant RNase H2 mice were intercrossed, no live homozygous mutant progeny were born, indicating that RNase H2 is essential. Further examination showed that homozygous mutant embryos existed in Mendelian ratios until embryonic day 10.5, after which they could no longer survive. The embryonic lethality presents a challenge to the study of RNase H2 mutants, and ideally conditional alleles can be generated so that the consequences of RNase H2 inactivation can be examined in adult mice. It is highly likely that RNase H2 is required at some key stage in development, so a conditional knockout mouse will help address the question of when exactly RNase H2 is needed.

AGS symptoms are clearly consequences of chronic exposure to elevated INF-α levels, which putatively in turn stem from faulty nucleic acid processing. Studies from yeast robustly show that RNase H2 inactivation leads to genomic instability, but it is unclear if (i) RNase H2 inactivation leads to genomic instability in human cells, (ii) genome instability contributes in any way to AGS or (iii) the genome instability caused by RNase H2 mutation could lead to any cancers. The AGS mouse model finally allowed for some genome instability assays to be performed in mammalian RNase H2 mutants. The cells from RNase H2 mutant mice had increased chromosomal abnormalities, activated checkpoints and increased phosphorylated H2AX foci, pointing to DNA damage (10). This demonstration of genome instability has important implications for understanding the RNase H2 function in mammals as well as its contribution to disease avoidance. There have been no direct connections between RNase H2 mutation and cancer, although a recent study found that *RNaseh2b* was overexpressed in adenocarcinoma tissue from gastric cancer (55). It will be intriguing to further explore sequencing data generated from tumor samples to identify any links between RNase H2 deficiency, genome instability and tumor development.

### Non-deleterious consequences of ribonucleotides in DNA

The increase in genome instability stemming from RNase H2 inactivation clearly is a negative outcome for the cell. However, if misincorporated ribonucleotides are truly so detrimental, perhaps DNA polymerases would have evolved more stringent mechanisms to keep rNTPs from getting into DNA, even when the rNTPs pools are so high. The fact that rNTP misincorporation persists and perhaps even is selected for argues that ribonucleotides in the genome can at times benefit the cell. Indeed, there are specific examples where ribonucleotides are performing a function by being in DNA, and it is likely that there are more, as yet undiscovered, instances of the same.

#### *Mating type switching in* Schizosaccharomyces pombe

Haploid cells of the fission yeast *S. pombe* exist in two distinct mating types dictated by the *mat1* locus. Ribonucleotide presence in the DNA has been shown to be important for mating type switching in *S. pombe* (56). Specifically, a two-ribonucleotide imprint directs the recombination that results in mating type switching. This imprint derives from failure to completely process the Okazaki fragment subsequent to DNA replication. Importantly, this provides an example of ribonucleotide-based epigenetic imprint that serves to differentiate sister chromatid strands, and could have implications for developmental processes in multi-cellular organisms.

#### Utilization of ribonucleotides during NHEJ repair

In humans, DNA polymerase mu (Polμ) is capable of using rNTPs instead of dNTPs during the non-homologous end-joining (NHEJ) process of double strand break repair (57). The X family polymerase Polμ has a very open ‘steric fence’ that controls sugar selection, with a glycine (G) residue in place of where the steric tyrosine (Y) residue is found in other X family polymerases Polβ and Polλ (58,59). This results in low sugar discrimination and hence, misincorporation of rNTPs during gap-filling and NHEJ. Surprisingly, this utilization of rNTPs as polymerase substrates has advantages. First, rNTPs are inserted with higher base fidelity than are dNTPs (57). Second, DNA ligase IV is stimulated by a 3′ terminal ribonucleotide; therefore, the insertion of rNTPs promotes NHEJ (60). Finally, as rNTPs are found in higher abundance than dNTPs, Polμ can utilize the more common substrate. The ability of this polymerase to be flexible with choice of substrates allows it to adapt to various conditions and maximize its repair fidelity and/or efficiency in response to nucleotide pool fluctuations. Similarly, bacterial and archaeal NHEJ pathways also use enzymes that insert ribonucleotides and preferentially ligate ends with a 3′ terminal ribonucleotide (61–63). The fact that this phenomenon is observed in all kingdoms of life suggests that ribonucleotide utilization during NHEJ is important, with ribonucleotides and/or their removal perhaps marking sites of junction or otherwise serving a purpose related to proper repair.

#### Signaling for MMR

MMR of base misincorporation errors made during DNA replication must be directed toward repair of the nascent strand using the template strand information in order to avoid mutation. As rNTP incorporation during replication marks the nascent strands, it has been suggested that the rNTPs may provide a mechanism to mark and initiate MMR in eukaryotes (3). While the lagging strand is discontinuous and has nicks from Okazaki fragments, the leading strand is largely intact. As this strand is replicated by Pol2 (Polϵ), which has a higher rNTP misincorporation rate than Pol3 (Polδ), nicking at embedded rNMPs by RNase H2 to initiate MMR is an attractive model.

Recent studies have made insightful connections between ribonucleotide misincorporation and MMR (64,65,27). The nicks introduced by RNase H2 act to enhance the efficiency of MMR. In bacterial systems, it is estimated that RNase H2 cleavage is responsible for directing around 10% of MMR (27). The MMR defect in RNase H2 mutants is enough to drive an increase in mutation rate, although the contribution of rNTPs to MMR is limited and clearly other factors are involved in directing MMR. Therefore, the creation of nicks by RNase H2 at ribonucleotides misincorporated into newly replicated DNA strands not only catalyzes the removal of the rNMP residue, but it also increases MMR, leading to fewer mutations. In this way, RNase H2 has dual protective mechanisms against mutation.

These examples lead to the question of whether having ribonucleotides in the genome is better than not having them, or if there is some optimal level of misincorporated ribonucleotides in the genome that strikes the right balance between being useful and being harmful. Intriguing experiments that test the fitness of cells with reduced ribonucleotide misincorporation can be performed to see if there is a disadvantage to having fewer ribonucleotides in the genome. Already there are polymerase mutants with more rigid gates that misincorporate 3X fewer ribonucleotides than the wild-type enzyme (41). Speculatively, one could engineer polymerases that were hyper-efficient at exonuclease proofreading, further reducing the stable misincorporation of ribonucleotides. Going even further, one could think about creating RNase H2 mutants that have increased enzymatic activity or altered localization that would increase the efficiency of ribonucleotide removal. The consequences of these changes on the fitness of the organism could then be studied over time, allowing for the extrapolation of the relative advantages and disadvantages of ribonucleotide misincorporation on a whole organism level.

## CONCLUSIONS

Misincorporated ribonucleotides alter the landscape of the genome in ways that are both beneficial and detrimental to the cell (Table 2). The stability of DNA renders it a good template for storing genetic information, so RNA contamination and resultant destabilization of DNA can compromise the fidelity of replication. However, semi-conservative replication generates identical copies of DNA strands, so the misincorporation of ribonucleotides provides a distinction between the older and the younger strand. The existence of this commonplace marker has implications for a host of things, including DNA repair, development and disease. A major outstanding question is where exactly in the genome are ribonucleotides being misincorporated. There is evidence that misincorporation is influenced by sequence context; however, no precise patterns have been characterized. Are repeat regions more vulnerable to ribonucleotide insertion? Genome-wide studies will surely shed light on this, as well as on the influence of chromatin state and transcriptional activity. Identification of any regions that are enriched for misincorporated ribonucleotides would give clues about possible functions, as will identification of regions with fewer misincorporated ribonucleotides. Cell- or tissue-specific patterns of ribonucleotide misincorporation would also be extremely fascinating to elucidate. In any case, ribonucleotide misincorporation and subsequent repair represent dynamic DNA transactions that could be subject to regulation and variation to meet changing requirements of the cell. The cost versus benefit of ribonucleotide misincorporation could fluctuate, and it will be interesting to learn what signals dictate this change in cost how the cell responds to it.

**Table 2.** Consequences of ribonucleotide misincorporation. Summary of studies showing negative or positive roles for ribonucleotide misincorporation.

**Consequences of ribonucleotide misincorporation**
**Negative**
Increased mutation rate (2,3,9,22,37,41)
Increased chromosomal abnormality (10,11,45)
Replication fork barrier (3,21,22)
Autoimmune disease in humans (12,49–51)
Embryonic lethal in mammals (10,11)
Cancer development?
**Positive**
Nascent strand discrimination to facilitate mismatch repair (4,64,65)
Mating type switching in *S. pombe* (56)
NHEJ pathway with Polμ (58–60)
